# Therapeutic Applications of Fibroblast Activation Protein (FAP)-Binding Radiopharmaceuticals: Review of Opportunities and Challenges

**DOI:** 10.3390/cancers17244019

**Published:** 2025-12-17

**Authors:** Justine Maes, Bernard Pôlet, Janke Kleynhans, Filip Van Herpe, Karolien Goffin, Jeroen Dekervel, Philippe Nafteux, Baki Topal, Frederik Cleeren, Christophe M. Deroose

**Affiliations:** 1 Nuclear Medicine and Molecular Imaging, Imaging and Pathology, KU Leuven, 3000 Leuven, Belgium; justine.maes@kuleuven.be (J.M.);; 2 Department of Nuclear Medicine, University Hospitals UZ Leuven, 3000 Leuven, Belgium; 3 Radiopharmaceutical Research, Department of Pharmaceutical and Pharmacological Sciences, KU Leuven, 3000 Leuven, Belgium; janke.kleynhans@kuleuven.be (J.K.);; 4 Department of Digestive Oncology, University Hospitals UZ Leuven, 3000 Leuven, Belgium; 5 Department of Thoracic Surgery, University Hospitals UZ Leuven, 3000 Leuven, Belgium; 6 Laboratory of Respiratory Diseases and Thoracic Surgery (BREATHE), Department of Chronic Diseases and Metabolism (CHROMETA), KU Leuven, 3000 Leuven, Belgium; 7 Department of Visceral Surgery, KU Leuven and University Hospitals UZ Leuven, 3000 Leuven, Belgium

**Keywords:** radioligand therapy, radionuclide therapy, FAP, FAPI, nuclear medicine, oncology

## Abstract

Fibroblast activation protein (FAP)-binding radiopharmaceuticals have emerged as promising candidates for both diagnostic and therapeutic applications in oncology due to their selective targeting of cancer-associated fibroblasts (CAFs). This review evaluates the current literature on the therapeutic use of FAP-targeted radiopharmaceuticals in human studies, with a focus on their safety, efficacy, and clinical applicability. Data on radionuclide type, clinical outcome, radiological and metabolic response and adverse events were extracted and summarized. The included studies demonstrated that lutetium-177,yttrium-90 and actinium-225 (in combination therapy) labeled FAP inhibitors exhibit high tumor uptake, with varying but mostly sufficient retention and a favorable safety profile. While mild adverse events such as fatigue, nausea and grade 1 or 2 hematotoxicity were observed, severe toxicities were rare. FAPI-based radionuclide therapies generally show high disease control rates, with promising results from tandem and combination strategies. The heterogeneity of tumor types and small sample sizes limited the generalizability of findings. FAP-targeted radioligand therapy appears to be a promising treatment option for patients with advanced cancer who have exhausted standard therapies. However, further large-scale, prospective clinical trials are necessary to determine optimal dosing strategies, long-term safety and efficacy across different tumor types. Emerging approaches, such as covalently binding FAP-targeted radiopharmaceuticals and the use of alpha-emitters such as actinium-225, lead-212 and bismuth-213, may further enhance treatment outcomes and warrant future investigation.

## Simple Summary

Fibroblast activation protein (FAP) is overexpressed in the tumor micro-environment in up to 90% of epithelial tumors and has limited expression in healthy tissues. Radionuclide therapy, using, for example, lutetium-177,yttrium-90 and actinium-225 (in combination therapy) labeled FAP-targeting radiopharmaceuticals, has shown promising results for therapeutic use through targeted radiation of FAP-expressing tumors. Emerging approaches include covalently binding FAP-targeted radiopharmaceuticals and the use of alpha-emitters. The aim of this review is to provide a comprehensive analysis and summary of the current literature on the therapeutic applications of FAP-binding radiopharmaceuticals in clinical studies. This includes investigating their therapeutic potential and safety profile and the challenges and opportunities that lie ahead. Overall, the treatments had a favorable safety profile and generally showed high disease control rates, with promising results from tandem and combination strategies.

## 1. Introduction

**Figure 1:**
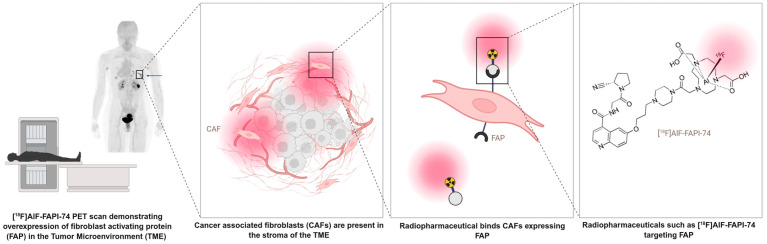
Schematic representation of FAP-targeting radiopharmaceuticals (Figure created using a licensed version of Biorender.com).

Cancer-associated fibroblasts (CAFs) are activated fibroblasts, commonly found in the tumor micro-environment of different tumor types and involved in facilitating tumor progression and dissemination. Fibroblast activation protein (FAP) is a membrane-bound serine protease that is highly expressed on the surface of these CAFs. FAP plays an important role in the proliferation of cancerous cells through remodeling of the tumor micro-environment, intracellular signaling, immunosuppression and stimulating angiogenesis and tumor growth. Hereby, they support tumor progression and the dissemination of malignant cells [1,2,3]. Expression of FAP has been reported in up to 90% of epithelial tumors [4]. Although fibroblasts are widely distributed throughout the body, in healthy tissue they generally exhibit little to no FAP expression. The limited expression of FAP in normal tissues and its pronounced overexpression in the tumor micro-environment makes it an interesting candidate for both diagnostic and therapeutic strategies. Targeting FAP with a radiolabeled FAP inhibitor (FAPI) allows for effective detection and potentially destruction of tumor tissue while minimizing the impact on healthy tissue [5]. The mechanism of action of radiolabeled FAP inhibitors is illustrated in Figure 1 [6].

The current literature demonstrates significant progress in the development and application of different FAP-binding radiopharmaceuticals, with varying biochemical and pharmacokinetic properties. Their dual role in both diagnostics and therapy makes FAP-binding radiopharmaceuticals promising candidates in cancer treatment, contributing to high accuracy in detecting cancer cells and offering personalized treatment options [7,8,9]. The class of diagnostic FAP-targeting radiopharmaceuticals labeled with gallium-68 (^68^Ga-FAPIs) has been shown to achieve a high tumor-to-background ratio (TBR) in various malignancies [5,10]. Radionuclide therapy (RNT), using, for example, lutetium-177 or yttrium-90 labeled FAP-targeting radiopharmaceuticals, has shown promising results in preclinical and clinical data for therapeutic use through targeted radiation of FAP-expressing tumors [8,11,12,13,14].

RNT offers several important advantages compared to conventional systemic treatments, particularly its ability to deliver targeted radiation to malignant tissues while sparing surrounding healthy organs. A key advantage is the generally favorable side-effect profile. Most patients tolerate treatment well, and serious adverse events are uncommon relative to chemotherapy or external-beam radiotherapy. Mild and transient symptoms such as fatigue or nausea may occur, but long-term toxicity is rare when appropriate eligibility criteria and posology based on dosimetry or clinical parameters are followed. Additionally, its minimally invasive administration (typically intravenous injection) and compatibility with outpatient care make it appealing for patients with advanced or metastatic disease. However, treatment efficacy can vary widely depending on heterogeneity of target expression, radiopharmaceutical biodistribution and patient-specific characteristics. Moreover, logistics related to radionuclide availability, regulatory requirements, specialized imaging and dosimetry infrastructure can limit widespread access. RNT represents a promising modality, but continued refinement in patient selection, radiopharmaceutical design and optimized posology regimens is needed to maximize therapeutic benefit while minimizing risk.

**Figure 2:**
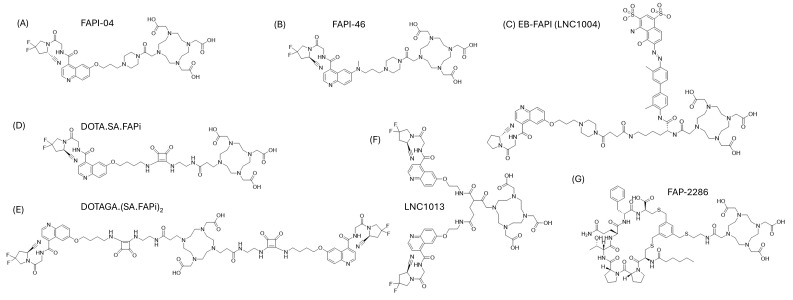
Chemical structures of FAP-targeting radiopharmaceuticals described in this review, including (**A**) FAPI-04 [8], (**B**) FAPI-46 [17], (**C**) EB-FAPI, (**D**) DOTA.SA.FAPi [40], (**E**) DOTAGA.(SA.FAPi)_2_ [40], (**F**) LNC1013 [41] and (**G**) FAP-2286 [40].

While the diagnostic application of FAP-binding radiopharmaceuticals is promising, the therapeutic application thereof remains more elusive. It is clear that the absorbed dose and effective half-life among various FAP-binding radiopharmaceuticals are different. The requirements for the ideal diagnostic radiopharmaceutical include high selectivity for the target and a fast circulatory clearance with high contrast at the time of imaging [15]. In such cases, small molecules like FAPI-04 (Figure 2) excelled, sometimes demonstrating a high tumor-to-background ratio even 10 min after administration [15]. The pharmacokinetic requirements for therapeutic radiopharmaceuticals are quite different, and often in direct contradiction with that of diagnostic radiopharmaceuticals. This includes potentially prolonged blood circulation, serum stability and persistent tumor retention. The aim is to maximize the effective tumor half-life and tumor radiation dose, with concurrent minimum radiation dose to healthy surrounding tissues. The first small molecules (e.g., FAPI-04) based on UAMC-1110 were developed as a method to reduce the molecular weight (from originally investigated antibodies), which improves tissue penetration and pharmacokinetics [15,16]. However, they demonstrated non-ideal tumor residency for therapy. Modification of the linker region between the quinoline moiety and chelator resulted in increased tumor uptake and improved pharmacokinetic properties, resulting in the establishment of FAPI-46 with a markedly longer tumor retention time compared to FAPI-04 [16,17]. These small molecules typically radiolabel efficiently with lutetium-177 or gallium-68, often with high radiochemical yields and high molar activities because of the straightforward DOTA-based coordination chemistry [15,16]. However, the radiochemical performance alone does not compensate for their short biological half-life in tumors. A case report presented that the tumor retention time of FAPI-46 might still be suboptimal, with washout recurring before the lutetium-177 has deposited the full radiation dose [18]. Various strategies have been applied to increase the tumor residency time of FAP-binding radiopharmaceuticals. The first strategy is to conjugate the FAP-binding moiety with an albumin binder, such as Evans Blue (EB) as demonstrated in the development of EB-FAPI [19]. Binding to albumin in circulation increases blood residency by slowing down elimination. It is hypothesized that prolonged presence in the blood will increase the concentration of the radiopharmaceutical at the tumor site. The second strategy commonly applied is to apply dimerization of the binding moiety, such as demonstrated with LNC1013 and DOTAGA.(SA.FAPi)_2_ [20,21]. It is hypothesized that dimerization increases the amount of radiopharmaceutical delivered to the tumor target and that these also represent higher chances of rebinding to the target, with slower off-rates compared to that of monovalent counterparts [22]. A third strategy reported in the literature is to replace the small molecule approach with that of a FAP-targeting peptide, for example, in FAP-2286, which consists of a cyclic peptide conjugated to the chelator 1,4,7,10-tetraazacyclododecane-1,4,7,10-tetraacetic acid (DOTA) [20]. A preclinical head-to-head comparison showed favorable results for the cyclic FAP-2286, with the highest TBR, durable uptake up to at least 72 h post-injection and the lowest uptake in healthy tissues (with the exception of the kidneys) [20]. Finally, ligands that lead to covalent bonds upon binding have recently been proposed as a strategy to increase tumor retention without the need for prolonged blood half-life, achieving irreversible target engagement and reporting up to a 13-fold increase in tumor retention compared to FAPI-04 [23]. These chemical strategies, though sometimes associated with more complex synthesis or altered molar activities, clearly illustrate the relationship between structural design and in vivo pharmacokinetics. The chemical structures of the radiopharmaceuticals described in this review are depicted in Figure 2.

The choice of radionuclide is another parameter to take into account. Lutetium-177 (^177^Lu) is currently one of the key radionuclides of choice in clinical practice, with, for example, [^177^Lu]Lu -PSMA-617 and [^177^Lu]Lu -DOTATATE gaining evidence in the treatment of prostate cancer and neuro-endocrine tumors, respectively. Lutetium-177 can be produced in large quantities and, with its relatively long half-life of 6.71 days, shipping to distant sites is possible. Moreover, the β^−^ particles have a favorable penetration range (average distance: 670 μm in soft tissue), and the concomitant emission of low-energy gamma photons enables post-therapy imaging and dosimetry [24,25]. As a pure β^−^ emitter, yttrium-90 (^90^Y) also has some established clinical applications. With a higher energy range, yttrium-90 has been suggested to be suited for more voluminous lesions. A shorter half-life (64 h) makes shipping challenging but offers options for vectors with shorter retention times [24,26]. Another radionuclide of interest is actinium-225 (^225^Ac). Emitting several α-particles in its decay, actinium-225 has a substantially higher linear-energy transfer than β^−^ emitters, hereby facilitating double-stranded DNA breaks. This allows effective cell death in targeted lesions, while minimizing damage to surrounding healthy tissue [27]. Other emerging radionuclides in FAPI RNT include astatine-211 (^211^At) [28,29,30,31], samarium-153 (^153^Sm) [32], iodine-131 (^131^I) [33,34], terbium-161 (^161^Tb) [35], lead-212 (^212^Pb) [36,37,38] and bismuth-213 (^213^Bi) [39]. A full summary of all the radionuclides applied in FAP-targeting therapy is provided in Table 1.

**Table 1:** Summary of radionuclides applied in the clinical setting as FAP-targeting therapeutic radiopharmaceuticals.

Radionuclide	Emission Characteristics	Range In Vivo (mm) [42,43]	Chelators Applied	FAP-Targeting Ligands	Design Features	Key Advantages	Limitations/Remarks	Reference
Beta-minus [or dual emissions]								
Lutetium-177	Emission of β- and γ Physical t½ = 6.65 days E_max_ = 498 keV	0.62–2.00	DOTA, DOTAGA	FAPI-04; FAPI-46; FAP-2286; LNC1013 EB-FAPI; DOTA.SA.FAPi DOTAGA.(SA.FAPi)_2_	Almost all design features have been implemented—monomeric, peptide, albumin binding and dimeric FAP vectors	Lutetium-177 offers easy logistics, moderate LET and can be used for comparison between design features.	Moderate energy might have less optimal penetration range for larger lesions. Lower LET.	[19,21,44,45,46,47,48,49,50,51,52]
Yttrium-90	Emission of β- Physical t½ = 64.1 h E_max_ = 2.3 MeV	5.30–12	DOTA	FAPI-46	Monomeric small molecule	High energy, useful for bulky lesions	Limited post-therapy imaging possibilities	[26,53,54]
Samarium-153	Emission of β- and γ Physical t½ = 46.7 h E_max_ = 806 keV	0.4	DOTA	FAPI-46	Monomeric small molecule	Readily available and inexpensive	Samarium-153 is hampered by low specific activity that can influence molar activity of final radiopharmaceuticals.	[32]
Iodine-131	Emission of β- and γ Physical t½ = 8.0 days E_max_ = 606 keV	0.80	Labeled through radioiododestannylation	FAPI-02 FAPI-04	Monomeric small molecule	Broad availability and inexpensive	Radiopharmaceuticals can be hampered by lower stability and off-target thyroid uptake.	[33,34]
Terbium-161	Emission of β- and conversion and Auger electrons Physical t½ = 6.9 days E_max_ = 590 keV	0.29	DOTAGA	DOTAGA.Glu.(FAPI)_2_	Dimeric small molecules	Same chemistry as lutetium-177; Auger and conversion electron emission might enhance microdosimetry	Still in preclinical development.	[35]
Alpha-emitters [or dual emissions]								
Actinium-225	α-particle emissions Physical t½ = 9.9 days E_max_ = 5.83–8.38 MeV, 4 α-emissions	0.04–0.10	DOTA DOTAGA	DOTAGA.FAPi FAPI-46	Monomeric small molecule	High LET, effective for micro-metastases and potential for large lesions since it can overcome mechanisms of radio-resistance to beta-minus emitters [55,56]	Possible daughter redistribution. Short range of α in vivo could hamper application in FAP. Limited production now (but with substantial industrial effort to increase future availability) [57].	[52]
Astatine-211	α-particle emissions Physical t½ = 7.2 h E_max_ = 8.5 MeV 1 α-emission	0.05	Aryl/borane conjugate	PDA-FAPI [At]FAPI_1-5_ APBA-FAPI	Monomeric small molecule, albumin binding	High LET, single alpha emission	Complex chemistry and weak supply chain. Short range of α in vivo could hamper application in FAP.	[28,29,30,31]
Lead-212	α-particle and β-emissions Physical t½ = 10.64 h E_max_ = 6.05–8.78 1 α-emission	<0.1	PSC (lead-specific chelator),	PSV-359	Structure not publicly declared	Allows for post-therapy imaging, high LET, in vivo alpha-particle generator.	Relatively novel, supply chain not established, and radiolabeling protocols still under development. Currently only preclinical application to FAP.	[36,37,38]
Bismuth-213	α-particle and γ emissions Physical t½ = 45.6 min E_max_ = 8.375 MeV 1 α-emission	0.05–0.10	DOTA	FAPI-46	Monomeric small molecule	Matches kinetics of FAP-targeting molecules with short biological half-life	Not readily available, logistical complications and short physical half-life. Short range of α in vivo could hamper application in FAP.	[39]

Radiobiological considerations are central to dictate the most optimal radionuclide and how radiation dose translates into therapeutic efficacy and safety. One consideration is the presence of activated fibroblasts within the tumor stroma, rather than the malignant cells themselves, except in some rare tumoral entities with direct FAP expression by the malignant cells [58]. It is therefore important to consider if the radionuclide would have the optimal range, or if crossfire effects can compensate for heterogenous target distribution. Additionally, the effect of immune system modulation needs to be considered as an additional factor, and alpha and beta-minus emitters have different tumor-specific effects within the tumor microenvironment [59]. Currently, there is no specific consensus about which type of therapeutic radiation would be most optimal for application in FAP-targeting.

The primary aim of this review is to provide a comprehensive analysis and summary of the current literature on the therapeutic applications of FAP-binding radiopharmaceuticals in clinical studies. This includes investigating their therapeutic potential and safety profile and the challenges and opportunities that lie ahead.

## 2. Results

An overview of relevant clinical experience is presented below, categorized by radionuclide and vector. Table 2 provides an overview of the selected studies, including the year of publication, number of participants, median age, sex ratio, radionuclide used and primary tumor type. Table 3 summarizes the clinical, biochemical, and radiological outcomes and the radiological and metabolic response according to RECIST and PERCIST criteria, respectively. Additionally, Table 4 details biochemical adverse events categorized according to CTCAE v5.0. Lastly, to summarize the advantages of different ligand design strategies, a quantitative comparison of key pharmacokinetic parameters for selected therapeutic ligands incorporating lutetium-177 is presented in Table 5.

### 2.1. [177. Lu]Lu-FAPI-04

In a retrospective analysis, four patients with progressive metastatic tumors (breast, thymic, thyroid cancer, ovarian carcinosarcoma) received a subtherapeutic dose of [^177^Lu]Lu-FAPI-04 (259–278 MBq) to evaluate its safety and therapeutic potential through gamma camera imaging [44]. The treatment was generally well-tolerated, with no severe toxicity reported. Although the highest absorbed dose in metastases was observed in bone lesions (0.62 ± 0.55 mGy/MBq), the overall radiation dose to tumor sites was relatively low, driven by relatively fast tumor washout. This suggested that further optimization, either by increasing the administered activity or by using higher-energy radionuclides with a shorter half-life, such as yttrium-90, may be needed to enhance therapeutic efficacy [44].

### 2.2. [177. Lu]Lu-FAP-2286

Baum et al. [45] retrospectively evaluated the cyclic FAP-binding radioligand in 11 patients with metastatic adenocarcinomas (pancreatic adenocarcinoma (*n* = 5); breast adenocarcinoma (*n* = 4); ovarian adenocarcinoma (*n* = 1) and rectal adenocarcinoma (*n* = 1)). One patient experienced grade 3 abdominal pain with vomiting, and one patient experienced grade 3 leukopenia. No grade 4 adverse events were reported (Table 4).

Significant improvement in pain scores was observed in three patients, and one patient reported improved physical capacity and quality of life. Post-therapy SPECT/CT images revealed significant tracer uptake and retention in tumoral lesions up to 10 days after injection, distinguishing this radioligand from [^177^Lu]Lu-FAPI-04. Response evaluation on morphological imaging (RECIST) showed two patients that exhibited stable disease (18%), while the remaining nine showed progressive disease 6 to 8 weeks after the first cycle, consistent with circulating tumor markers.

**Figure 3:**
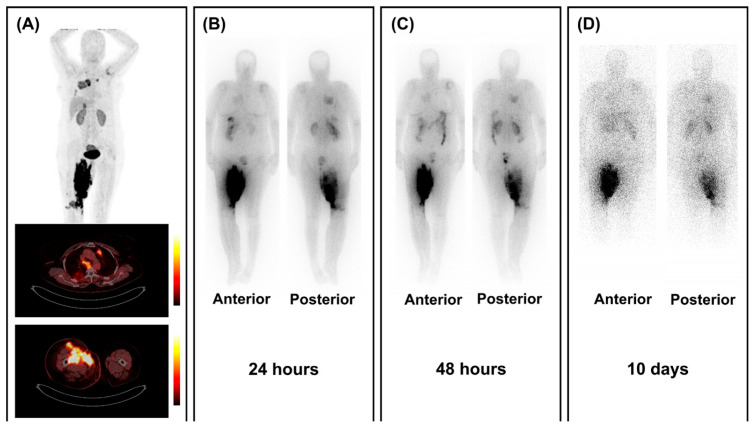
Retention of [^177^Lu]Lu-FAPI-2286 in tumoral lesions. (**A**) [^68^Ga]Ga-FAP-2286 PET/CT images show high FAP expression in a patient with lung-metastasized sarcoma localized in the right inguinal area. (**B**–**D**) Post-treatment scintigraphy images show uptake of [^177^Lu]Lu-FAPI-2286 in all lesions up to 10 days after RNT with 7.4 GBq of [^177^Lu]Lu-FAPI-2286. Figure reproduced from Banihashemian et al., Feasibility and therapeutic potential of [^177^Lu]Lu-FAPI-2286 in patients with advanced metastatic sarcoma, Eur J Nucl Med Mol Imaging.2024 Dec, doi: 10.1007/s00259-024-06795-7 [46]. under CC BY 4.0 (https://creativecommons.org/licenses/by/4.0/, accessed on 3 September 2025).

Following the promising diagnostic [^68^Ga]Ga-FAP-2286 study [61] by Banihashemian et al., eight sarcoma patients were included for [^177^Lu]Lu-FAP-2286 therapy [46]. All patients had unresectable disease or were progressive after conventional treatments. Two patients passed away due to unrelated events (myocardial infarction and suicide), before starting therapy and after three cycles, respectively, and one patient’s treatment was stopped after two cycles due to progressive disease. Five patients completed four cycles of RNT (6660–7400 MBq, q6–8 weeks). No grade 3 or 4 side effects were reported. Post-therapy SPECT/CT images showed tumor retention up to 10 days after treatment (see Figure 3). Response assessment showed a 52.4% reduction in the average volume of the primary tumor, along with a significant decrease in the SUV_max_ (30%) and TBR (44%) of metastatic lesions, particularly in cases involving lung metastases. In addition to improved physical capacity, patients also reported a marked reduction in pain and greater satisfaction with the treatment [46].

These trials demonstrate that [^177^Lu]Lu-FAP-2286 has a favorable safety profile, with manageable side effects and long tumor retention. Although varying morphological response was observed, there was an overall good clinical response to the treatment.

### 2.3. [177. Lu]Lu-EB-FAPI ([^177^Lu]Lu-LNC1004)

In a non-randomized, activity-escalation, open-label study of [^177^Lu]Lu-EB-FAPI, 12 patients with metastatic radioiodine-refractory (RAI-R) thyroid carcinoma were treated [19]. Patients were divided into three groups: Group A (*n* = 3) received 2.28 ± 0.10 GBq, Group B (*n* = 6) 3.50 ± 0.09 GBq, and Group C (*n* = 3) 4.80 ± 0.28 GBq. Post-therapy imaging showed significant uptake and prolonged retention in tumors up until 7 days post injection. A longer effective half-life was observed in bone metastases compared to lymph node and other metastatic sites.

**Figure 4:**
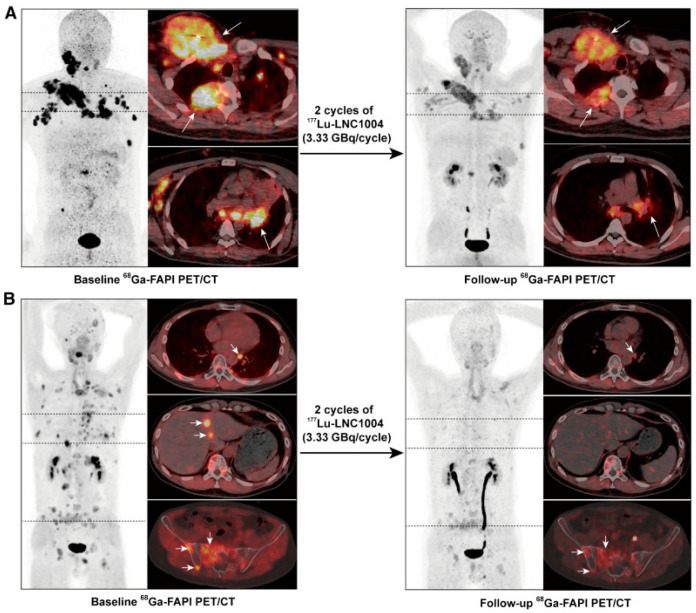
Two cases with partial response after two cycles of 3.33 GBq [^177^Lu]Lu-EB-FAPI. At baseline, ^68^Ga-FAPI PET/CT shows high FAP expression in metastatic lesions (bone, lymph nodes, liver) in two patients with disease progression after TKI treatment for RAI-R thyroid cancer. (**A**): a 36-year-old man with high uptake in metastatic lymph nodes and bone metastases (left, arrows). (**B**): a 42-year-old man with high uptake in bone, liver and lymph node metastases (left, arrows) Follow-up ^68^Ga-FAPI PET/CT indicates a favorable response with reduced FAPI uptake and size reduction (right panels, arrows). Figure reproduced from Fu et al., Fibroblast Activation Protein-Targeted Radioligand Therapy with 177Lu-EB-FAPI for Metastatic Radioiodine-Refractory Thyroid Cancer: First-in-Human, Dose-Escalation Study, Clin Cancer Res. 2023 Dec 1, doi: 10.1158/1078-0432.CCR-23-1983. [19] under CC BY 4.0 (https://creativecommons.org/licenses/by/4.0/, accessed on 3 September 2025).

Group-specific findings include the following: in Group A, one patient experienced mild transient thrombocytopenia and neutropenia after the first cycle. In Group B, two patients reported increased pain at bone metastases lasting up to 6 days. One patient required medical intervention for mild thrombocytopenia and leukopenia. Group C exhibited more severe hematotoxicity, including grade 3–4 thrombocytopenia and leukopenia, leading to reduction of injected activity and cessation of further activity escalation. Three patients showed reduced tumor metabolism (from group B, see Figure 4), while others had stable disease (*n* = 7) or progression (*n* = 2) [19].

Following this activity-escalating study, 28 patients with end-stage metastatic disease, predominantly RAI-R thyroid cancer (*n* = 13), were treated with 3.33 GBq/cycle [47]. Most patients received two cycles (*n* = 19), 10 patients received three, and six patients received four cycles. Post-therapy imaging revealed tumoral uptake up to 2 weeks after injections, indicating a favorable tumor retention. Reported toxicity was rather high: 19 patients (68%) experienced grade 3 or 4 adverse events, of whom 12 (43%) had hematotoxicity (Table 4). However, the authors attribute this to the lutetium-177-therapy only in six patients. Other suspected causes were advanced tumor stage and extensive pretreatment. Response rates were evaluated with RECIST, showing partial response in 20% and stable disease in 45% of patients [47].

Three case reports describe favorable outcomes using combination therapy with [^177^Lu]Lu-FAP-2286. The first describes a 56-year-old man with metastasized lung adenocarcinoma who was eligible for targeted therapy due to EGFR exon 19 deletion and therefore received combination therapy of 80 mg Osimertinib with 7.4 GBq [^177^Lu]Lu-FAP-2286 [62]. Significant improvement in dyspnea was observed 2 weeks later, and imaging showed reduced uptake in the tumor lesions. The brain metastasis completely disappeared, supporting the good therapeutic effect of the combination therapy [62]. Another case report described the treatment of a 42-year-old woman with metastatic ductal breast carcinoma using the combination of chemotherapy (TCHP; Docetaxel, Carboplatin, Trastuzumab and Pertuzumab) and four cycles of 6.66 GBq [^177^Lu]Lu-FAP-2286 [63]. A complete therapeutic response was observed, with the disappearance of [^18^F]FDG and [^68^Ga]Ga-FAP-2286 uptake in all previously identified lesions using PET/CT [63]. The third combination therapy included sorafenib (tyrosine kinase inhibitor) with [^177^Lu]Lu-FAPI-2286 in a 48-year-old man with medullary thyroid carcinoma [64]. The patient received four cycles (cumulative dose of 29.6 GBq, q4 months). Good clinical and biochemical response was observed with a significant decrease in serum calcitonin and CEA [64].

Some case reports also underscore the possible significance of [^177^Lu]Lu-FAP-2286 therapy in palliative pain management. A 55-year-old man with advanced end-stage melanoma reported a significant reduction in pain after one cycle of [^177^Lu]Lu-FAP-2286, after which he progressed [65]. Two women with bone-metastasized invasive ductal carcinoma were treated with 7.4 GBq [^177^Lu]Lu-FAP-2286, with a significant reduction in pain lasting up to 120 days after therapy. One patient experienced grade 3 thrombocytopenia, otherwise no adverse events were reported [66].

Other reports on the use of [^177^Lu]Lu-FAP-2286 in metastasized settings include (i) squamous cell carcinoma of the lung (7 GBq) with reduced uptake on follow-up [^68^Ga]Ga-FAP-2286 PET/CT in a 59-year-old man [67]; (ii) bladder carcinoma (7.4 GBq) in a 73-year-old patient with symptom improvement and a decrease in SUV_max_ (from 5.4 to 2.4 at the primary bladder lesion) [68]; (iii) rhabdoid meningioma grade 3 (7.4 GBq) in a 43-year-old patient with a decrease in number of lesions and SUV_max_ (9.6 to 5.2) in liver lesions [69]; (iv) leiomyosarcoma in a 67-year-old man (four cycles; cumulative activity of 32.9 GBq) with mixed response [70]; (v) solitary fibrous tumor in a 57-year-old woman (7.4 GBq) with a reduction in number of metastases and tracer uptake after 12 weeks [71]; (vi) nasopharyngeal cancer in a 24-year-old (7.4 GBq) with progressive disease after two cycles [72]; (vii) RAI-R papillary thyroid carcinoma in a 38-year-old man (7.4 GBq) resulting in a good clinical response and a decline in thyroglobulin levels [73]; (viii) mediastinal sarcoma in a 67-year-old man (four cycles, cumulative dose 23 GBq) with decreased uptake and size of the metastatic lesions [70].

None of these case reports reported significant side effects.

### 2.4. [177. Lu]Lu-LNC1013

Tan et al. developed a novel FAPI dimer, [^68^Ga]Ga-LNC1013, with high tumoral uptake in gastrointestinal cancers [41]. Three patients with metastatic gastric cancer were subsequently enrolled in a dosimetric analysis for [^177^Lu]Lu-LNC1013 [48]. They received a single injection of 1.86–2.04 GBq. Post-therapy imaging revealed lesion uptake until 48 h, but significantly decreased at later timepoints (96 and 168 h), indicating a shorter retention time than for other ligands. No adverse events were reported. The highest mean absorbed doses in target organs were observed in the thyroid and pancreas (respectively 1.82 Gy/GBq and 0.44 Gy/GBq), compatible with the physiological uptake seen with pretherapeutic [^68^Ga]Ga-LNC1013 PET/CT. High physiological thyroid uptake has been demonstrated with other FAP-targeting radiopharmaceuticals, in particular, dimeric ones [74]. Tumoral lesions had a mean absorbed dose of 0.34 Gy/GBq with [^177^Lu]Lu-LNC1013 [48].

### 2.5. [177. Lu]Lu-FAPI-46

In a preliminary study [49], the dosimetry, safety and feasibility of [^177^Lu]Lu-FAPI-46 were evaluated in 21 patients with advanced cancer. Nineteen patients showed significant FAP uptake and were included in an activity escalation protocol, receiving activities ranging from 1.4 GBq to 4.44 GBq (median of 3.7 GBq/cycle, q4–6 weeks).

The treatment was generally well-tolerated, with one patient (sarcoma) experiencing grade 3 anemia, grade 1 thrombocytopenia and grade 1 leukopenia, likely due to concomitant chemotherapy, according to the authors. One patient also reported increased bone pain. No dose-related toxicity was observed, and dosimetric analysis in 11 patients showed minimal uptake in normal tissues, supporting the safety of the treatment. Twelve patients showed stable disease (67%) and six exhibited progressive disease (one patient died before receiving the treatment).

[^177^Lu]Lu-FAPI-46 has also been reported to be efficient in palliative pain management. Administration of 7.4 GBq in a patient with multiple endocrine neoplasia type 2A syndrome, diagnosed with a paraganglioma in the sacrum, medullary thyroid carcinoma and bilateral pheochromocytoma, resulted in resolution of abdominal pain [75]. A 34-year-old man with RAI-R thyroid carcinoma [76] was treated with four cycles of cumulative 22.2 GBq, showing improved pain scores and an improved performance and resulting in stable disease.

Two other reports describe transient amelioration of the pain and mixed response: one cycle of 1.85 GBq in a 52-year-old woman with metastatic adenocarcinoma of the ampulla of Vater resulted in an initial pain reduction. However, after 2 weeks, the pain as well as tumor markers increased again [18]. In a 25-year-old patient with metastatic nasopharyngeal carcinoma, a reduction in bone pain was noted after administration of 3.7 GBq. Follow-up after 8 weeks showed a mixed response with regression of lesions in the ribs and thoracic vertebrae but an increase in other lesions. These results might be attributed to the lower administered activity in these patients.

### 2.6. [177. Lu]Lu-DOTA.SA.FAPi and [^177^Lu]Lu-DOTAGA.(SA.FAPi)_2_

In 10 patients with various solid tumors, the biodistribution and dosimetry of [^177^Lu]Lu-DOTA.SA.FAPi (*n* = 3, breast carcinoma) and [^177^Lu]Lu-DOTAGA.(SA.FAPi)_2_ (*n* = 7, paraganglioma and thyroid carcinoma) were evaluated. Patients in the [^177^Lu]Lu-DOTA.SA.FAPi group (group 1) received a median administered activity of 2.96 GBq in one cycle, while those in the [^177^Lu]Lu-DOTAGA.(SA.FAPi)_2_ group (group 2) received two cycles with a median activity of 1.48 GBq in the first cycle. Both groups exhibited good tolerance with no early adverse effects post-administration [50].

Group 1 initially showed a response to the experimental treatment; however, clinical relapse of symptoms was observed after 6 weeks, and two participants died. In contrast, group 2 demonstrated clinical response in all patients with no deaths reported. Both radiopharmaceuticals were well-tolerated with minimal toxicity. One patient with pre-existing grade 1 anemia presented with grade 3 anemia and grade 1 thrombocytopenia after administration.

[^177^Lu]Lu-DOTAGA.(SA.FAPi)_2_ showed significantly longer retention of the radiotracer and higher absorbed dose in the tumor, albeit with higher uptake in the colon and kidneys, yet still maintained good tolerance compared to [^177^Lu]Lu-DOTA.SA.FAPi.

A second retrospective study [21] focused on the safety and efficacy of [^177^Lu]Lu-DOTAGA.FAPi dimer therapy in 19 patients with metastatic breast cancer. These patients, who exhibited progressive disease under standard treatment, underwent therapy with [^177^Lu]Lu-DOTAGA.(SA.FAPi)_2_ and [^177^Lu]Lu-DOTAGA.Glu.(FAPi)_2_. A total of 65 cycles were administered with a median activity of 5.5 GBq per cycle (mean cumulative administered activity 19 ± 5.7 GBq). Sixteen of the 19 patients underwent follow-up imaging with [^68^Ga]Ga-DOTA.SA.FAPi PET/CT, where four of the 16 showed partial response, six stable disease, and six showed disease progression(Table 3).

The treatment was well-tolerated, with no grade 3 or 4 toxicity observed. Additionally, significant clinical response was noted, including improvement in pain scores in over 90% of participants as assessed by the visual analog score (VAS), suggesting that this therapy has the potential to improve the quality of life for patients by significantly reducing pain without severe toxicity.

The same group evaluated treatment with [^177^Lu]Lu-DOTAGA.(SA.FAPi)_2_ in 15 patients with RAI-R metastasized thyroid cancer [51]. Nine patients received three cycles, three patients received four cycles, and three patients two cycles, with a mean cumulative activity of 8.2 ± 2.7 GBq. No grade 3 or 4 toxicities were observed. Biochemical response (decrease in thyroglobulin levels) was observed in all patients. Interim [^68^Ga]Ga-DOTA.SA.FAPi PET/CT in seven patients showed stable disease in three patients and partial response in four (not included in Table 3 since it is not mentioned that PERCIST criteria were used). Clinical response, assessed with VAS and global pain assessment (GPA) response criteria, was favorable, with an overall response rate of 92% [51].

**Figure 5:**
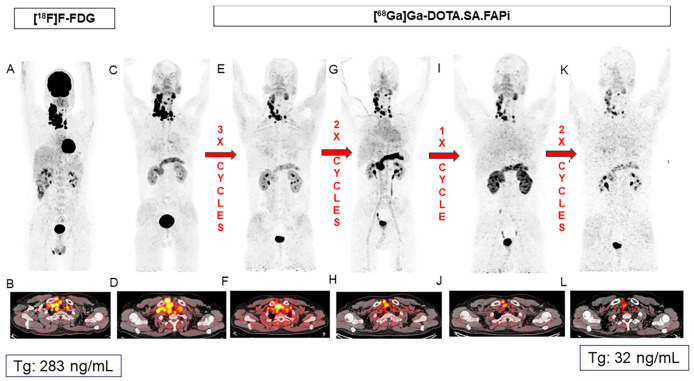
Favorable response after combined [^177^Lu]Lu- and [^225^Ac]Ac-DOTAGA.FAPi dimer therapy in a 37-year-old patient with RAI-R thyroid cancer. Baseline PET/CTs ([^18^F]FDG (**A**,**B**) and [^68^Ga]Ga-DOTA.SA.FAPi (**C**,**D**)) show intense tracer uptake in the malignant mass in the right lobe of the thyroid and in the lymph node metastases. Patient received eight cycles of FAPI RNT, including five cycles of [^177^Lu]Lu- and three cycles of [^225^Ac]Ac-DOTAGA.FAPi. Post-therapy PET/CT images (**E**–**L**) show favorable treatment response. Patient was eligible for surgery after completing all cycles. Figure reproduced from Ballal et al., Long-Term Outcomes in Radioiodine-Resistant Follicular Cell-Derived Thyroid Cancers Treated with [177Lu]Lu-DOTAGA.FAPi Dimer Therapy. Thyroid. 2025 Feb; doi: 10.1089/thy.2024.0229 [52] under CC BY 4.0 (https://creativecommons.org/licenses/by/4.0/, accessed on 3 September 2025).

Based on these promising results, Ballal et al. investigated the long-term outcomes of [^177^Lu]Lu-DOTAGA.(SA.FAPi)_2_ therapy (median of 5.5 GBq/cycle, q8 weeks) in 73 patients with RAI-R follicular cell-derived thyroid cancers [52]. Grade 3 anemia and thrombocytopenia occurred in four (5.4%) and three (4%) patients, respectively. Interestingly, eight patients were treated with combined [^177^Lu]Lu- and [^225^Ac]Ac-DOTAGA.FAPi dimer therapy (median of 7.7 MBq/cycle for ^225^Ac, see Figure 5). This subgroup had a tendency for higher OS and PFS (median not attained for both), compared to 32 and 29 months in the [^177^Lu]Lu-DOTAGA.(SA.FAPi)_2_-only group (median follow-up of 3 years). Response assessment per PERCIST criteria in 36 patients showed partial response in 50%, stable disease in 25% and progression in the remaining 25%.

These studies underscore the therapeutic potential of ^177^Lu-labeled DOTAGA-FAPi dimers, with excellent clinical response rates of more than 90% and disease control rates of up to 75%, and promising results for combined lutetium-177 and actinium-225 therapy.

The group of Ballal et al. also published case reports on [^177^Lu]Lu-DOTA.SA.FAPi (3.2 GBq) [77], the first in-human experience with [^177^Lu]Lu-DOTAGA.(SA.FAPi)_2_ (1.65 GBq) [78] and [^177^Lu]Lu-DOTAGA.Glu.(FAPi)_2_ (two cycles of 7.4 GBq) [79], respectively, in end-stage breast cancer, medullary thyroid carcinoma and glioblastoma multiforme. A good clinical response was observed in all patients, with a substantial reduction of the masses of the thyroid carcinoma and glioblastoma multiforme.

### 2.7. [90. Y]Y-FAPI-46

This FAP-binding radioligand was evaluated in a single-center, retrospective study [53] involving 21 patients, primarily with metastatic sarcoma (*n* = 16) and with pancreatic, prostate and gastric cancer. Patients underwent a total of 47 cycles. RECIST disease control was observed in eight of the 21 patients. PERCIST disease control, reflecting stable metabolic disease, was noted in eight of the 21 patients, including seven sarcoma patients and one patient with another solid tumor (not specified). The median PFS was 3.4 months, and the median OS was 10 months.

Grade 3/4 anemia was observed in six of the 21 patients, and grade 3 and 4 thrombocytopenia was found in four and two patients, respectively. According to the authors, the thrombocytopenia was related to the FAPI RNT in only four out of six patients. The other grade 3/4 adverse events were deemed related to disease progression.

In a single-center, retrospective study involving nine patients treated with [^90^Y]Y-FAPI-46 [26], good tolerance was observed along with initial signs of therapeutic efficacy. Six patients were treated for metastatic soft tissue or bone sarcoma, and three patients for pancreatic carcinoma. Two patients received a starting activity of 7.4 GBq, while the other seven patients received 3.8 GBq. The median dose for subsequent cycles was 7.4 GBq. When indicated, these cycles were administered in two fractions of 3.8 GBq (fractionated to optimize radiation due to the short biological half-life). One patient underwent three cycles, and two other patients underwent two cycles. The remaining six patients underwent one cycle due to very limited radiotracer uptake (*n* = 2) or due to deterioration or death before the second cycle could be administered (*n* = 4).

No acute toxicity was observed following radiopharmaceutical administration. During follow-up (median follow-up time of 44 days), four patients exhibited grade 3 thrombocytopenia, possibly related to the administration of [^90^Y]Y-FAPI-46, but also chronologically related to either tumor progression or the initiation of concomitant systemic therapy. Other observed side effects, such as grade 3 anemia and grade 3 or higher elevations in liver or pancreatobiliary serum markers, were related to disease progression (Table 4).

Radiological disease control was observed in 50% of the participants, with one patient (11%) even showing tumor regression after the first cycle. In terms of metabolic response, two of seven patients (29%) exhibited disease control, and other patients showed progressive disease.

**Figure 6:**
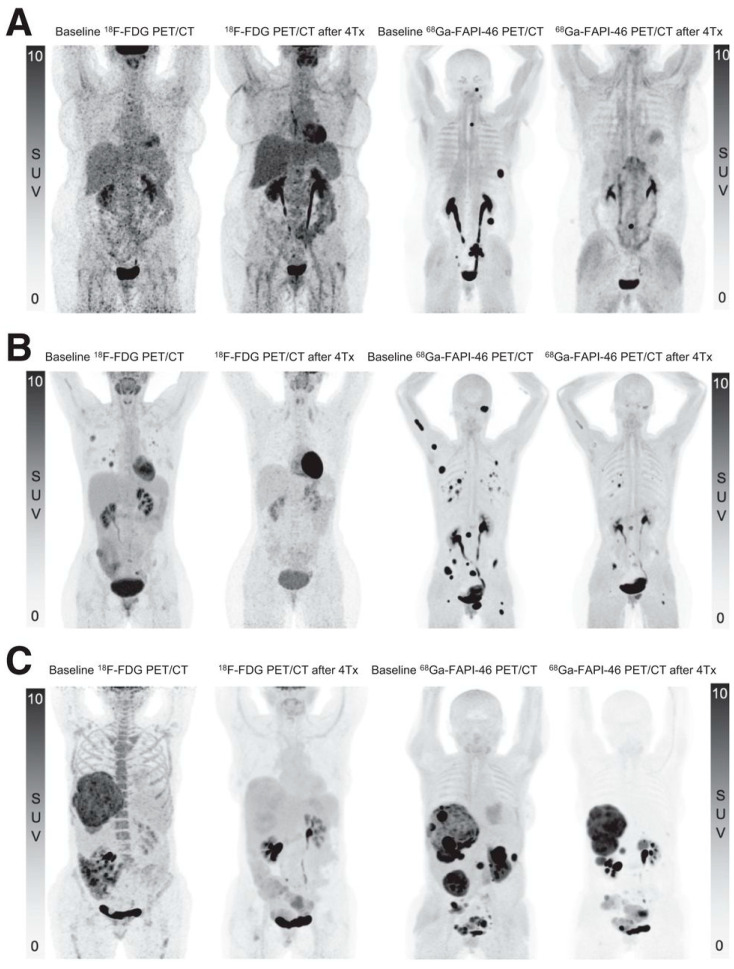
Near-complete metabolic response in three patients with solitary fibrous tumors (SFT). Baseline and follow-up PET/CT MIP images ([^18^F]FDG and [^68^Ga]Ga-FAPI-46) after four cycles of [^90^Y]Y-FAPI-46 in three patients with SFT. (**A**) A 58-year-old woman with primary retroperitoneal SFT, progressive after conventional treatments with lymph node and soft tissue metastases. (**B**) A 38-year-old woman with lung, lymph node, peritoneal and bone metastases. (**C**) A 61-year-old woman with liver and peritoneal metastases. Figure reproduced from Lanzafame et al., ^90^Y-FAPI-46 Theranostics Leads to Near-Complete Metabolic Response in 3 Patients with Solitary Fibrous Tumors. J Nucl Med. 2025 Sep 2; doi: 10.2967/jnumed.125.269572 [54]. under CC BY 4.0 (https://creativecommons.org/licenses/by/4.0/, accessed on 3 September 2025).

Lanzafame et al. recently published promising findings in three patients with advanced solitary fibrous tumors [54]. After establishing high FAP expression with messenger RNA and protein expression of FAP on biopsies, patients received 3.7 GBq [^90^Y]Y-FAPI-46 during the first cycle and 7.4 GBq during the following three cycles (q4.5 weeks). Besides grade 1 thrombocytopenia in one patient, no toxicities were observed. RECIST response criteria showed partial response in two patients and stable disease in one, while PERCIST criteria indicated complete metabolic response in one and partial response in another patient (one not eligible, see Figure 6). Clinical response was also favorable, with resolution of fatigue and pain in two patients [54].

To address the short tumor retention time of FAPI-46, Kratochwil et al. [32] labeled it with ^153^Sm (half-life of 46.3 h) to treat a patient with lung-metastasized soft tissue sarcoma. Three cycles (cumulative 20 GBq) of [^153^Sm]Sm-FAPI-46 were combined with 8 GBq of [^90^Y]Y-FAPI-46, because of a low specific activity of [^153^Sm]Sm-FAPI-46. This resulted in stable disease up to 8 months. Despite the good therapeutic response, the authors acknowledge the current practical difficulties with ^153^Sm and suggest the use of alternative isotopes [32]. Following this report, a patient with concomitant metastasized breast and colorectal cancer received four cycles of 7.4 GBq [^90^Y]Y-FAPI-46 in combination with palbociclib. There was a remission of the lesions related to the colorectal cancer and stable disease regarding the breast cancer lesions lasting 7 months [80].

### 2.8. [213. Bi]Bi-FAPI-46

To address the relatively short tumor retention time of FAPI-46, Helisch et al. combined this vector molecule with the short half-life α-emitter ^213^Bi and administered therapy in a fractionated scheme [39]. Six patients with progressive disease after conventional treatments (colon adenocarcinoma (*n* = 2), triple-negative breast adenocarcinoma, anal squamous cell carcinoma, PSMA-negative prostate adenocarcinoma and signet ring cell colon carcinoma) received a mean of 1.6 GBq [^213^Bi]Bi-FAPI-46. This was administered in 5–12 fractions per patient over a period of up to 107 h per patient, which presented some practical challenges. No toxicities were observed. After follow-up, one patient had partial response (17%), one stable disease, and the other four progressive disease (based on visual assessment; these results were not included in Table 3 since it is not mentioned that PERCIST criteria were used).

## 3. Discussion

The results in this review demonstrate that FAP-binding radiopharmaceuticals, such as lutetium-177 and yttrium-90-based therapies, are promising for the treatment of various types of cancer, particularly in patients who have exhausted other treatment options. Overall, the treatments had favorable safety profiles, with mostly limited grade 1 or 2 side effects such as headache, nausea, anemia and thrombocytopenia. More severe grade 3 or 4 hematotoxicity was observed in approximately 5% of patients treated with [^177^Lu]Lu-DOTAGA.(SA.FAPi)_2_ and, more commonly, in up to 40% of patients treated with [^177^Lu]Lu-EB-FAPI or [^90^Y]Y-FAPI-46 [19,47,52].

The uptake of radiopharmaceuticals in tumor tissues was high, with low uptake in healthy tissues (see Table 3 and Table 5). Only [^177^Lu]Lu-FAPI-04 showed lower tracer uptake in tumor lesions, making this radioligand less attractive for therapeutic purposes. Fast wash-out in tumoral lesions was also observed with [^177^Lu]Lu-LNC1013, along with high uptake in healthy thyroid tissue. Furthermore, although promising, practical issues were a big limitation in the treatment with ^153^Sm-labeled FAPIs and in the regimen of fractionated ^213^Bi-labeled FAPIs that need to be addressed.

Across the available FAP-binding radiopharmaceuticals, several molecular attributes consistently correlate with therapeutic potential. The most promising candidates combine high tumor uptake with prolonged intratumoral retention, low background accumulation, and radiochemical stability compatible with therapeutic radionuclides. Original small monomeric molecules show minimal off-target uptake and short blood residency, leading to a favorable safety profile but sub-par tumor residency. Peptide-based FAP-binding radiopharmaceuticals such as FAP-2286 demonstrate sustained retention for up to 10 days with short systemic exposure, translating into favorable clinical responses in both adenocarcinoma and sarcoma cohorts [45,46,61]. Dimeric FAP-binding radiopharmaceuticals such as DOTAGA.(SA.FAPi)_2_ likewise show substantially longer tumor residence and higher absorbed tumor doses than monomeric analogues [21,50,51,52], with corresponding improvements in clinical outcomes. Albumin-binding variants like EB-FAPI enhance systemic exposure and increase the tumor input, leading to higher tumor dose deposition, though often at the cost of increased hematotoxicity [19,47]. The ideal FAP-binding radiopharmaceutical would have a high affinity for FAP, selectivity and prolonged intratumoral retention. Furthermore, it is likely that the molecular size would be somewhat larger than that of the small molecules (either a peptide or a dimeric radiopharmaceutical) to enhance tumor retention or use a small molecule with covalent bond generation. The currently available FAP-binding radiopharmaceuticals are based on beta-minus emitter radionuclides and will most likely be administered in a combination setting with other therapeutic strategies (e.g., chemotherapy) and not as monotherapy. Radiopharmaceuticals with long tumor retention and/or alpha-emitters might be studied in a monotherapy setting as well as in combination therapy.

Disease control rates were mostly high, ranging from 62.5% to 75% with the use of [^177^Lu]Lu-DOTAGA-FAPi dimers [21,52], 65% to 83% with [^177^Lu]Lu-EB-FAPI [19,47], 67% for [^177^Lu]Lu-FAPI-46 [49] and up to 100% with [^90^Y]Y-FAPI-46 [54]. One report in advanced adenocarcinomas treated with [^177^Lu]Lu-FAPI-2286 only had a disease control rate of 18% [45], whereas the trial in sarcoma patients observed a disease control rate of 80% [46]. Strategies to further improve outcomes include tandem treatment regimens, where the concomitant use of [^177^Lu]Lu- and [^225^Ac]Ac-DOTAGA.FAPi therapy showed promising results, with higher OS and PFS in eight patients with RAI-R follicular cell-derived thyroid cancer, compared to single use of [^177^Lu]Lu-DOTAGA.FAPi dimer therapy [52]. Another strategy, implemented in some case reports, is combination therapy of FAPI RNT with immunotherapy, chemotherapy or other targeted therapies [62,63,64]. To confirm that these combination therapies have an additional benefit, these results must be verified in larger trials. Overall, excellent clinical response was reported, with some case reports also underlining the possible use of FAPI RNT in palliative pain treatment.

This review provided an overview of the current literature regarding FAP-binding radioligands in human studies. The literature is still limited to retrospective case series and phase I studies, but the initial results are very promising, particularly concerning the safety of these radiopharmaceuticals. However, efficacy is variable, with room for improvement. New techniques, such as the use of radiopharmaceuticals that generate covalent bonds upon receptor binding, offer groundbreaking potential for future research. The study by Cui et al. [23], using a sulfur (VI) fluoride exchange chemistry-based linker, demonstrated low exposure to healthy tissue and, due to covalent binding, 257% more tumor uptake than the original FAPI-04 ligand, with 13-fold higher tumor retention (observed by integrating the area under the curve of the time–activity curves). Additionally, they showed therapeutic effects in mouse models, including nearly complete tumor regression. This strategy leads to irreversible binding to targeted proteins such as FAP and could be broadly applied in the field of RNT. This study could represent a potential paradigm shift in this field. While covalent ligand design represents one notable innovation with potential clinical translatability, it must be acknowledged that it is one of several emerging strategies aimed at improving FAPI RLT that have been studied in a preclinical setting [81,82].

This overview highlights the potential of these radiopharmaceuticals and their relevance for patients with limited therapeutic options. Covalent targeted radiotherapy and the promising results of combination therapies demonstrate that there is still significant potential to enhance the efficacy of FAP-targeted therapy.

The limitations of the current evidence base regarding FAPI RNT and this review must also be considered. Firstly, the studies involved only represent a small number of participants, ranging from a minimum of three to a maximum of 73 patients. Consequently, the results may not be representative of a broader population, making it difficult to detect small effects. Another limitation is the variability in posology regimens, which can lead to inconsistent results. Most of the studies discussed here are retrospective in nature. The heterogeneity of the tumor types is also a significant limitation, as they may exhibit varying responses to the same treatment, making it challenging to compare treatments across patients. The extreme variability in radiopharmaceutical vectors, with vastly different tumor uptake and retention profiles, as well as the wide variety of radionuclides applied, induces high variety in the quality and quantity of radiation dose received at the tumor site. All patients discussed in this review had run out of validated treatment options, which may lead to an underestimation of the results in patients at an earlier point in their disease evolution. Finally, the lack of control groups is a major limitation.

Despite significant progress, numerous challenges remain, such as the heterogeneity of FAP expression between different tumor types and even within a single patient, including intralesional heterogeneity, interlesional heterogeneity and temporal heterogeneity, and the influence of FAPI RNT on FAP expression. This variability can limit the universal application of both diagnostic and therapeutic FAP-binding radiopharmaceuticals. Additionally, there is a need for larger, prospective, randomized clinical trials, and longer follow-up periods are required to monitor long-term safety and efficacy.

## 4. Conclusions

In summary, these FAP-targeted approaches show promising potential for patients with limited treatment options. The high tumor uptake, low toxicity, and preliminary efficacy of these treatments make them compelling candidates for further clinical development. Large-scale clinical studies are essential to confirm long-term safety, optimal dosing strategies, and broader applicability of these treatments. Additionally, ongoing research into the covalent binding of FAP with radiopharmaceuticals could further enhance the effectiveness of radionuclide therapy, potentially leading to significant advancements in cancer treatment.

## Figures and Tables

**Table 2:** Summary of Included Studies: Participant Characteristics, Radiopharmaceutical Utilized, and Primary Tumors.

Author	Year	Participants (*n*)	Median Age in Years (Range)	Sex Ratio (M/F)	Radiopharmaceutical	Primary Tumor
Kuyumcu et al. [44]	2021	4	61 (41–73)	1/3	[^177^Lu]Lu-FAPI-04	Metastatic advanced-stage cancer (breast, thymic, thyroid cancer, ovarian carcinosarcoma)
Baum et al. [45]	2022	11	61 (40–78)	5/6	[^177^Lu]Lu-FAP-2286	Advanced adenocarcinomas (pancreas, breast, rectum and ovary cancer)
Banihashemian et al. [46]	2024	8 *	60.5 (17–75)	6/2	[^177^Lu]Lu-FAP-2286	Advanced metastatic sarcoma
Fu et al. [19]	2023	12	52.5 (32–72)	8/4	[^177^Lu]Lu-EB-FAPI	Thyroid cancer
Fu et al. [47]	2025	28	56 (43–67)	15/13	[^177^Lu]Lu-EB-FAPI	Metastatic advanced-stage cancer: Radioiodine-refractory thyroid cancer (*n* = 13), breast cancer (*n* = 3), neuroendocrine carcinoma (*n* = 2), sarcoma (*n* = 2), non–small cell lung cancer (*n* = 2), gastric cancer (*n* = 1), colon cancer (*n* = 1), nasopharyngeal cancer (*n* = 1), renal cancer (*n* = 1), esophageal cancer (*n* = 1), neuroendocrine prostate cancer (*n* = 1)
Wang et al. [48]	2025	3	(67–76)	3/0	[^177^Lu]Lu-LNC1013	Metastatic gastric cancer
Assadi et al. [49]	2021	21	50 (6–79)	10/11	[^177^Lu]Lu-FAPI-46	Advanced cancer (ovarian, sarcoma, breast, rectum, colon, lung, pancreas, prostate, bile duct, thyroid and cervical cancer)
Ballal et al. [50]	2021	10	^177^Lu-DOTA.SA.FAPi: 50 (31–63) ^177^Lu-DOTAGA.(SA.FAPi)_2_: 51 (26–63)	4/6	[^177^Lu]Lu-DOTA.SA.FAPi and [^177^Lu]Lu-DOTAGA.(SA.FAPi)_2_	Breast cancer, thyroid cancer and paraganglioma
Yadav et al. [21]	2024	19	46 (30–70)	1/18	[^177^Lu]Lu-DOTAGA.(SA.FAPi)_2_ and [^177^Lu]Lu-DOTAGA.Glu.(FAPi)_2_	Breast cancer
Ballal et al. [51]	2022	15	57 (39–67)	4/11	[^177^Lu]Lu-DOTAGA.(SA.FAPi)_2_	Radioiodine-refractory metastasized thyroid cancer
Ballal et al. [52]	2025	73	Mean age 54.3 (27–80)	36/37	[^177^Lu]Lu-DOTAGA.(SA.FAPi)_2_ (*n* = 65) and [^177^Lu]Lu- and [^225^Ac]Ac-DOTAGA.FAPI dimers (*n* = 8)	Radioiodine-resistant follicular cell-derived thyroid cancers
Fendler et al. [53]	2022	21	61 (22–83)	8/13	[^90^Y]Y-FAPI-46	Sarcoma (*n* = 16), pancreatic (*n* = 3), prostate (*n* = 1) and gastric cancer (*n* = 1)
Ferdinandus et al. [26]	2022	9	57 (22–66)	4/5	[^90^Y]Y-FAPI-46	Sarcoma and pancreatic cancer
Lanzafame et al. [26]	2025	3	58 (38–61)	0/3	[^90^Y]Y-FAPI-46	Advanced solitary fibrous tumor
Helisch et al. [39]	2024	6	47 (16–77)	2/4	[^213^Bi]Bi-FAPI-46	Colon adenocarcinoma (*n* = 2), triple-negative breast adenocarcinoma, anal squamous cell carcinoma, PSMA-negative prostate adenocarcinoma and signet ring cell colon carcinoma

* One patient died before receiving the first cycle of FAPI RNT.

**Table 3:** Summary of Clinical, Biochemical and Survival Outcomes and Radiological and Metabolic Response as per RECIST and PERCIST Criteria, Respectively.

Author (Year)	Median FU	Median Cycles FAP RNT (*n*)	Mean Absorbed Dose (Gy/GBq)	Clinical Response (*n*)	RECIST Response	PERCIST Response	Median PFS in Months (OS in Months)
Kuyumcu et al. (2021) [44]	One week after last imaging study	1	Bone metastases: 0.62 Metastatic lymph nodes: 0.38 Liver metastases: 0.33 Metastatic soft tissue: 0.37 Kidneys: 0.25 Bone marrow: 0.04	NR	NR	NR	NR
Baum et al. (2022) [45]	Until death or disease progression	2	Bone metastases: 3.0 Liver metastases: 0.4 Kidneys: 1.0 Bone marrow: 0.05	Pain reduction (3/11)	SD: 2/11(18%) PD: 9/11 (82%)	NR	2 (6)
Banihashemian et al. (2024) [46]	NR	4	NR	Improvement in physical capacity (7/7) Reduction in pain (2/7)	PR: 4/5 (80%) ^1^ PD: 1/5 (20%) ^1^	NR	NR
Fu et al. (2023) [19]	6 weeks after second cycle	2	Bone metastases: 5.12 Metastatic lymph nodes: 5.95 Other metastases: 15.27 Kidneys: 1.32 Bone marrow: 0.11	NR	PR: 3/12 (25%) SD: 7/12 (58%) PD: 2/12 (17%)	NR	NR
Fu et al. (2025) [47]	18.1 months	2	Primary tumor lesions: 4.69 Bone metastases: 4.57 Metastatic lymph nodes: 4.88 Lung metastases: 6.47 Other metastases: 3.77	NR	PR: 4/20 (20%) ^2^ SD: 9/20 (45%) ^2^ PD: 7/20 (35%) ^2^	NR	4 (6.3)
Wang et al. (2025) [48]	3 months	1	Tumor lesions: 0.34 Kidneys: 1 Bone marrow: 0.05 Thyroid: 1.82 Pancreas: 0.44	NR	NR	NR	NR
Assadi et al. (2021) [49]	Until death or disease progression	2	NR	No change in ECOG (Eastern Cooperative Oncology Group) or KPS (Karnofsky Performance Scale)	SD: 12/18 (67%) PD: 6/18 (33%)	NR	3.0 (4.0)
Ballal et al. (2021) [50]	4.5 months	[^177^Lu]Lu-DOTA.SA.FAPi: 1 [^177^Lu]Lu-DOTAGA.(SA.FAPi)_2_: 2	[^177^Lu]Lu -DOTA.SA.FAPi and ^177^Lu-DOTAGA.(SA.FAPi)_2_ Kidneys: 0.68 and 3.74 Bone marrow: <0.01 and 0.02 Median absorbed dose in tumor lesions: 0.603 and 6.70	Clinical response in all patients treated with [^177^Lu]Lu-DOTAGA.(SA.FAPi)_2_ (7/10)	NR	NR	NR
Yadav et al. (2024) [21]	14 months	3	NR	VAS response criteria: 26.3% (5/19) complete response, 15.7% (3/9) partial response, 42% (8/19) minimal response, 11% (2/19) stable disease, 5% (1/19) no response.	NR	PR: 4/16 (25%) ^3^ SD: 6/16 (37.5%) ^3^ PD: 6/16 (37.5%) ^3^	8.5 (12)
Ballal et al. (2022) [51]	7.4 months	3	Tumor lesions: 10.8	VAS and GPA response criteria: 23% (3/13) complete response, 38.4% (5/13) partial response, 30.7 (4/13) minimal response, 7.7% (1/13) no response.	NR	NR	NR
Ballal et al. (2025) [52]	3 years	4	NR	NR	NR	PR: 18/36 (50%) ^4^ SD: 9/36 (25%) ^4^ PD: 9/36 (25%) ^4^	29 (32)
Fendler et al. (2022) [53]	Until 18 months or death	2–3	Lesion 1: 2.81 Lesion 2: 2.15	NR	PR: 1/16 (6%) ^5^ SD: 7/16 (44%) ^5^ PD: 8/16 (50%) ^5^	SD: 8/15 (53%) ^5^ PD: 6/15 (40%) ^5^	3.4 (10)
Ferdinandus et al. (2021) [53] Ferdinandus et al. (2022) [28]	44 days (IQR, 36–83.5 days)	1	Lesion 1: 1.28 Lesion 2: 0.95	NR	SD: 4/8 (50%) ^6^ PD: 4/8 (50%) ^6^	PR: 1/7 (14.3%) ^6^ SD: 1/7 (14.3%) ^6^ PD: 5/7 (71.4%) ^6^	NR
Lanzafame et al. (2025) [54]	NR	4	Reported per patient: Tumor lesions: 2.92, 2.25 and 1.94 Kidneys: 0.55, 0.23 and 0.27 Bone marrow: 0.02, 0.04 and 0.03	Resolution of fatigue and abdominal pain (2)	PR: 2/3 (66%) SD: 1/3 (33%)	CR: 1/2 (50%) ^7^ PR: 1/2 (50%) ^7^	NR
Helisch et al. [39]	NR	1 fractionated cycle (range 5–12 fractions over up to 107 h)	NR	NR	NR	NR	NR

**^1^** Only five of eight patients underwent RECIST evaluation; ^2^ Only 20 of 28 patients underwent RECIST evaluation; **^3^** Only 16 of 19 patients underwent PERCIST evaluation; ^4^ Only 36 of 73 patients underwent PERCIST evaluation; ^5^ Only 16 of 21 patients underwent RECIST evaluation, and 15 of 21 patients PERCIST evaluation; ^6^ Only eight of nine patients underwent RECIST evaluation, and seven of nine patients PERCIST evaluation. ^7^ Only two of three patients underwent PERCIST evaluation. NR = not reported, CR = complete response, PR = partial response, SD = stable disease, PD = progressive disease [19,21,39,44,45,46,47,48,49,50,51,52,53,54].

**Table 4:** Summary of Reported Biochemical Adverse Events (CTCAE v5.0).

Author (Year)	Anemia (*n*/Total)	Leukopenia (*n*/Total)	Thrombocytopenia (*n*/Total)	Nephrotoxicity (*n*/Total)	Other (*n*)
Kuyumcu et al. (2021) [44]	None (until 10 days after injection)	None (until 10 days after injection)	None (until 10 days after injection)	None (until 10 days after injection)	None (until 10 days after injection)
Baum et al. (2022) [45]	G1: 5/11 after C1, 3/8 after C2 G2: 3/11 after C1, 4/8 after C2 No G3 or G4	G1: 1/11 after C1 G2: 2/11 after C1, 2/8 after C2 G3: 1/8 after C2 No G4	G1: 3/11 after C1, 5/8 after C2 No G2, G3 or G4	G2: 1/11 after C1, 1/8 after C2 No G3 or G4	Headache (*n* = 5); Grade 3 abdominal pain, nausea and vomiting (*n* = 1)
Banihashemian et al. (2024) [46]	No G3 or G4	No G3 or G4	No G3 or G4	No G3 or G4	None
Fu et al. (2023) [19]	G1: 1/3 after C1 (group A), 1/6 after C1, 2/6 after C2 (group B). G2: 1/6 after C1, 1/6 after C2 (group B). No G3 or G4	G1: 1/3 after C2 (group A), 1/6 after C1, 3/6 after C2 (group B). G2: 1/6 after C1, 1/6 after C2 (group B). G3: 1/3 after C1 (group C).	G1: 1/3 after C1, 1/3 after C2 (group A), 3/6 after C1 (group B), 1/3 after C1 (group C). G2: 2/6 after C2 (group B). G3: 1/3 after C2 (group C). G4: 1/6 after C2 (group B), 1/3 after C1 (group C)	NR	Neutropenia, hypoalbuminemia
Fu et al. (2025) * [47]	24/28 with 6/28 G3 or G4 **0/28**	14/28 with 2/28 G3 or G4 **2/28**	22/28 with 8/28 G3 or G4 **4/28**	None	Neutropenia G3 or G4 (*n* = 2), tumor pain (*n* = 2)
Wang et al. (2025) [48]	No clinically relevant changes	No clinically relevant changes	No clinically relevant changes	No clinically relevant changes	None
Assadi et al. (2021) [49]	G3: 1 **/21	G1: 1 **/21	G1: 1 **/21	None	NR
Ballal et al. (2021) [50]	G3: 1/10	None	G1: 1/10	NR	NR
Yadav et al. (2024) [21]	G1: 2/19 G2: 1/19	None	G1: 1/19 G2: 2/19	None	NR
Ballal et al. (2022) [51]	None	None	None	None	Grade 1 diarrhea (*n* = 1), fatigue (*n* = 3)
Ballal et al. (2025) [52]	G3: 4/73	NR	G3: 3/73	NR	Pleural effusion (*n* = 2)
Fendler et al. (2022) [53]	G1-2: 5/21 G3-4: 6/21 ***	G1-2: 5/21 ***	G1-2: 5/21 *** G3-4: 6/21 ****	G1-2: 3 ***	Neutropenia ***
Ferdinandus et al. (2022) [26]	G1-2: 2/9 G3: 1/9	G1-2: 1/9	G1-2: 2/9 G3: 4/9	G1-2: 3/9	Neutropenia
Lanzafame et al. (2025) [54]	None	None	G1: 1/3	None	None
Helisch et al. (2024) [39]	None	None	None	None	None

* Numbers related to RNT according to authors are marked in bold. Other suspected causes were advanced tumor stage and extensive pretreatment. ** Patient received concomitant chemotherapy. *** According to authors, not related to the radioligand therapy but to disease progression. **** Only four of six related to the radioligand therapy; the other two were deemed related to disease progression. NR = not reported, G = grade, C = cycle.

**Table 5:** Quantitative Comparison of Key Pharmacokinetic Parameters for Selected Therapeutic Ligands Incorporating Lutetium-177—A Comparison of Ligand Design Strategies.

Radiopharmaceutical (Radionuclide)	Design Feature	Tumor Retention/Effective Half-Life (Qualitative)	Mean Absorbed Dose to Tumor Lesions (Gy/GBq)	References
[^177^Lu]Lu-FAPI-04	Monomeric small-molecule FAPI	Rapid tumor washout; relatively low tumor dose despite imaging signal up to several days post-injection	Bone metastases: 0.62 ± 0.55; metastatic lymph nodes: 0.38 ± 0.22; liver metastases: 0.33 ± 0.21; soft-tissue metastases: 0.37 ± 0.29	Kuyumcu et al. [44]
[^177^Lu]Lu-FAPI-46	Monomeric small-molecule FAPI	Intermediate retention; short biological half-life noted as a rationale for using shorter-lived radionuclides in some protocols	NR (clinical study provides organ dosimetry and safety; lesion-level Gy/GBq not reported)	Assadi et al. [49]
[^177^Lu]Lu-FAP-2286	Cyclic peptide	High and durable lesion uptake with visible retention up to 10 days post-therapy	Significant uptake and long tumor retention reported with a high absorbed dose to tumors; 3.0 ± 2.7 (range 0.5–10.6).	Baum et al. [45]
[^177^Lu]Lu-EB-FAPI	Albumin-binding small molecule	Prolonged tumor uptake up to 7 days in the dose-escalation study and up to ≈2 weeks in the expanded cohort; longer effective half-life in bone metastases than in lymph nodes and other lesions. Effective blood half-life (0.21 ± 0.11 h, half-life α; 68.01 ± 26.69 half-life β).	Primary lesions: 4.69 ± 3.83; bone metastasis: 4.57 ± 1.98; lymph node metastasis: 4.88 ± 4.39; lung metastases: 6.47 ± 6.75; other metastases: 3.77 ± 1.74.	Fu et al. [47] Wang et al., 2025 [60]
[^177^Lu]Lu-DOTAGA.(SA.FAPi)_2_	Dimeric FAP ligand	Significantly longer tumor retention and higher tumor dose than DOTA.SA.FAPi; durable uptake across cycles	Median absorbed dose in tumor lesions: 6.70 in initial series; up to 10.8 Gy/GBq in thyroid cancer cohort	Ballal et al. [21,50,51,52]
